# Supplementary material for: Conceptual Framework of a Psychotherapeutic Consultation in the Workplace: A Qualitative Study
Source: Int J Environ Res Public Health. 2022 Nov 12;19(22):14894. doi: 10.3390/ijerph192214894 (PMC9690206; doi:10.3390/ijerph192214894)
Supplement: Supplementary file 1 [file ijerph-19-14894-s001.zip › ijerph-1990896-supplementary.pdf]

Supplement S1 – Interview guidelines for dealing with therapists/practitioners

Motivation and expectations			
Interview partner	Key questions	Check whether this has been addressed!	Questions in detail
External psychotherapists	Why did you decide to implement PSIW?	Accessibility?<br>Personal motivation?	Why are you participating in PSIW?<br>What is the most interesting part for you?
	What did you **expect** from this offer?	What kind of patients?<br>Procedure?<br>Expectations at all?<br>Outcome?<br>Acceptance within the company?	Did you have concerns about it?

Work in detail			
	Key questions	Check whether this has been addressed!	Questions in detail
External psychotherapists	In what way differs your therapy when working in a company compared to working in a hospital (**Experience** Part 1)?	Time constraints<br>Methods<br>Structure of therapy session<br>Interventions	
External psychotherapists	Do you believe that PSIW focuses on different topics?<br>Could you explain in more detail please? (**Experience** Part 2)	Reaching different and more patients<br>Acute vs. chronic problems<br>Professional vs. private problems	Have you been well prepared for the topics that have risen up?
External psychotherapists	Did the offer meet your needs?	Place	Do you think you got close to your patient?<br><br>Would you have needed more time?<br><br>What could have been done better?

Supplement S2 – Interview guidelines for dealing with patients

Motivation and expectations			
Interview partner	Key questions	Check whether this has been addressed!	Questions in detail
Service user	What made you participate in PSIW (**motivation**)?	Flow of information<br>Thought about it for a longer time	Would you have entered psychosomatic treatment somewhere else if PSIW would not have been offered? Could you explain in more detail please?<br><br>Has it been easier to take advantage of the offer within the company instead of looking for something else outside of the company?
Service user	What did you **expect** from the offer?	Pre-understanding<br>First thoughts<br>Procedure	Did you have concerns about it?<br>What benefits did you wish for regarding the offer?

Work in detail			
	Key questions	Check whether this has been addressed!	Questions in detail
	What **experience** have you gathered while taking part in the offer?	Communication in the company<br>Personal thoughts	Have your expectations been met?
Service user	Did the offer meet your **expectations**?	Procedure<br>Place	Did anything change due to PSIW?<br>What are you taking with you from this offer?<br>What could have been done better?

Supplement S3 – Interview guidelines for organizers in a company

Motivation and expectations			
Interview partner	Key questions	Check whether this has been addressed!	Questions in detail
Company health promotion team	In which way are you participating in the offer and how did you get involved in it?<br>**(motivation)**		What is your personal motivation?<br>Which role did you play?
	What did/do you expect from the offer?	What should be included in the offer?<br>Place	What do you expect from the offer?<br>Did you have concerns?

Work in detail			
Who?	Key questions	Check whether this has been addressed!	Questions in detail
Company health promotion team	How about your experiences regarding the offer?	Feedback /atmosphere<br>Topic of discussion in the company<br>Cooperation with different actors	Have your expectations been met?<br>Did you personally talk to people and recommend the offer?
Company health promotion team	Are you satisfied with the offer?		What could have been done better?
